# Determining the individual relationship between the step width and peak knee adduction moment during stepping in medial knee osteoarthritis

**DOI:** 10.1016/j.clinbiomech.2025.106619

**Published:** 2025-07-22

**Authors:** Raziyeh Baghi, Wei Yin, Subham Badhyal, Ahmed Ramadan, Giovanni Oppizzi, Zongpan Li, Peter Bowman, Frank Henn, Li-Qun Zhang

**Affiliations:** a Department of Physical Therapy & Rehabilitation Science, University of Maryland Baltimore, Baltimore, MD, United States; b Fischell Department of Bioengineering, University of Maryland College Park, College Park, MD, United States; c Department of Orthopaedics, University of Maryland Baltimore, Baltimore, MD, United States; 1 School of Applied Engineering and Technology, New Jersey Institute of Technology.; 2 Phoenix Children’s Hospital, Phoenix, AZ, United States.; 3 University of Arizona College of Medicine-Phoenix, Phoenix, AZ, United States.; 4 Office of Science and Engineering Laboratories, Center for Devices and Radiological Health, Food and Drug Administration, Seattle, WA, United States.

**Keywords:** Biomechanics, Kinematics and kinetics, Knee osteoarthritis, Rehabilitation, Knee adduction moment

## Abstract

**Background:**

Step-width modification can reduce peak knee adduction moment during gait in individuals with knee osteoarthritis, but determining optimal subject-specific step-width without testing multiple discrete positions remains a clinical challenge.

**Method:**

We investigated step-width’s relationship with peak knee adduction moment in 14 individuals with medial knee osteoarthritis and 14 healthy controls using a robotic stepping system with motorized footplates moving between narrow, neutral, and wide step-widths. We analyzed peak knee adduction moment-step width relationship slopes and compared peak three-dimensional knee moments between all stepping conditions using repeated-measure ANOVA analysis.

**Findings:**

Both groups showed negative peak knee adduction moment-step width slopes, indicating reduced peak knee adduction moment with wider step-widths (knee osteoarthritis: *P* = 0.019, Controls: *P* = 0.016), with knee osteoarthritis group showing significantly higher slope and intercept values (*P* < 0.01, *P* < 0.001). Both groups demonstrated lower peak knee adduction moment and knee adduction moment impulse with wide step-width versus narrow and neutral step-widths (all *P* < 0.001). Lower peak knee adduction moment during wide step-width significantly correlated with increased tibia medial tilt (*P* < 0.001), increased footplate lateral reaction force (*P* = 0.023), reduced footplate inversion reaction torque (*P* < 0.001), reduced stepping speed (*P* = 0.022) and absence of knee osteoarthritis (*P* < 0.001).

**Interpretation:**

Wider step-width effectively reduces peak knee adduction moment and knee adduction moment impulse during stepping. The robotic stepping system enables precise subject-specific step-width determination using peak knee adduction moment-step width relationships, potentially offering individualized rehabilitation strategies for knee osteoarthritis management.

## 1. Introduction

Knee osteoarthritis (KOA), a prevalent joint disorder affecting the elderly, is a significant contributor to global disability (Quintana et al., 2008). It disproportionately impacts the medial knee compartment, which is more susceptible to osteoarthritis (OA) than the lateral compartment, often leading to pain and decreased mobility (Hernborg and Nilsson, 1977; Wise et al., 2015). Key to understanding and managing KOA is the knee adduction moment (KAM), which is the moment acting on the knee in the frontal plane and a surrogate measure of the load distribution across the medial compartment of the knee joint (Kutzner et al., 2013). Peak knee adduction moment (pKAM) and knee adduction moment impulse (ImpKAM) magnitude have been linked to the development, severity, and progression of medial KOA (Miyazaki et al., 2002; Thorp et al., 2010).

Gait retraining has been considered a potential conservative treatment option for KOA (Bennett et al., 2017; Hunt et al., 2018; Hunt and Takacs, 2014; Shull et al., 2013). Gait modification strategies such as altering step-width have proven effective in reducing KAM during various activities, including level walking and stair climbing (Fregly et al., 2007; Guo et al., 2007). Additionally, they have been shown to improve knee function and decrease pain. For instance, Bennett et al. demonstrated that increasing step-width by 0.06 m resulted in a 9.7 % reduction in pKAM during level walking (Bennett et al., 2017). However, individual responses to these strategies vary significantly, with some studies reporting pKAM reductions ranging from 3 % to 30 % (Favre et al., 2016; Gerbrands et al., 2017; Simic et al., 2011). Collectively, these investigators have suggested personalized gait retraining approaches to reduce variability in response to KAM reduction and optimize outcomes for individuals with KOA. Some of the previous studies assessed more personalized strategies, such as using 26 % and 39 % of participants’ leg length as wide and wider step-width, respectively (Bennett et al., 2017; Paquette et al., 2014a; Paquette et al., 2014b) or habitual, halved, double, and triple step-widths (Stief et al., 2021). Additionally, Xu et al. presented a personalized multi-parameter gait retraining approach using mapping-based dosage determination with real-time biofeedback to modify foot progression angle (FPA) and step-width (Xu et al., 2020). While this study manipulated a variety of parameters within a wide range of motion, it did not evaluate KAM during uniform increments of change in each of these gait parameters, omitting potentially ideal intermediate widths that could induce the desired KAM reduction without causing an unnatural walking pattern (Favre et al., 2016; Gerbrands et al., 2017). Studying uniform increments could provide valuable insights into the smallest modification in each parameter that brings about a meaningful reduction in KAM. However, these strategies limit step-width modifications to discrete conditions.

Although previous studies have demonstrated that personalized step-width can reduce KAM, these investigations have primarily relied on discrete step-width conditions and laboratory-based motion analysis systems. Such approaches may overlook optimal intermediate step-widths that could yield greater KAM reductions while preserving natural gait patterns. In the present study, we employed a robotic elliptical stepping system, which allows for smooth, continuous motion of the footplates in transverse and frontal planes (off-axis planes). This innovative approach enables precise manipulation and measurement of step-width while analyzing KAM in real-time and provides an immediate estimation of the subject-specific step-width for each individual (Kang et al., 2014; Kang et al., 2019; Lee et al., 2020; Ren et al., 2013). By capturing data across a continuous range of step-widths, our study aims to identify personalized gait modifications that achieve targeted reduction of KAM while minimizing alterations of other gait parameters and maintaining patient comfort. The proposed method utilizes an inexpensive goniometer to assess knee moment in real-time without needing a motion analysis laboratory and post-processing techniques. Therefore, the primary purpose of this study was to examine knee moments and ImpKAM in individuals with medial KOA and age and gender-matched healthy controls during elliptical stepping while dynamically varying the footplate positions between narrow, neutral, and wide step-widths. We hypothesized that increasing step-width would reduce pKAM and ImpKAM without affecting peak knee flexion moment (pKFM) and peak knee internal rotation moment (pKIRM). Additionally, we hypothesized that the slope of the relationship between step-width and KAM would be different from zero and could predict subject-specific step-width modifications for targeted KAM reductions.

## 2. Materials and methods

### 2.1. Participants

Fourteen individuals with medial KOA and fourteen age (±5 years) and gender-matched healthy controls without lower limb pain and musculoskeletal injury within the past six months participated in this study (Table 1). The inclusion criteria for the KOA group were the presence of pain in the medial compartment of the right knee more than one day per week in the last six weeks (Shull et al., 2013) and radiographic evidence of medial KOA with Kellgren & Lawrance (K/L) grade higher than 1 that was obtained from the participant’s medical record. The exclusion criteria for both groups included body mass higher than 136 kg (as a safety limit for the elliptical system), intra-articular pain relief injection or corticosteroid use within the prior six months, knee or hip arthroplasty surgery on either side, neurological conditions, severe cardiovascular disease, and uncontrolled hypertension. This study was approved by the institutional review board (IRB) of the University of Maryland Baltimore, USA, and all participants signed an informed consent form approved by the IRB.

### 2.2. Experimental procedure

**Fig. 1.**
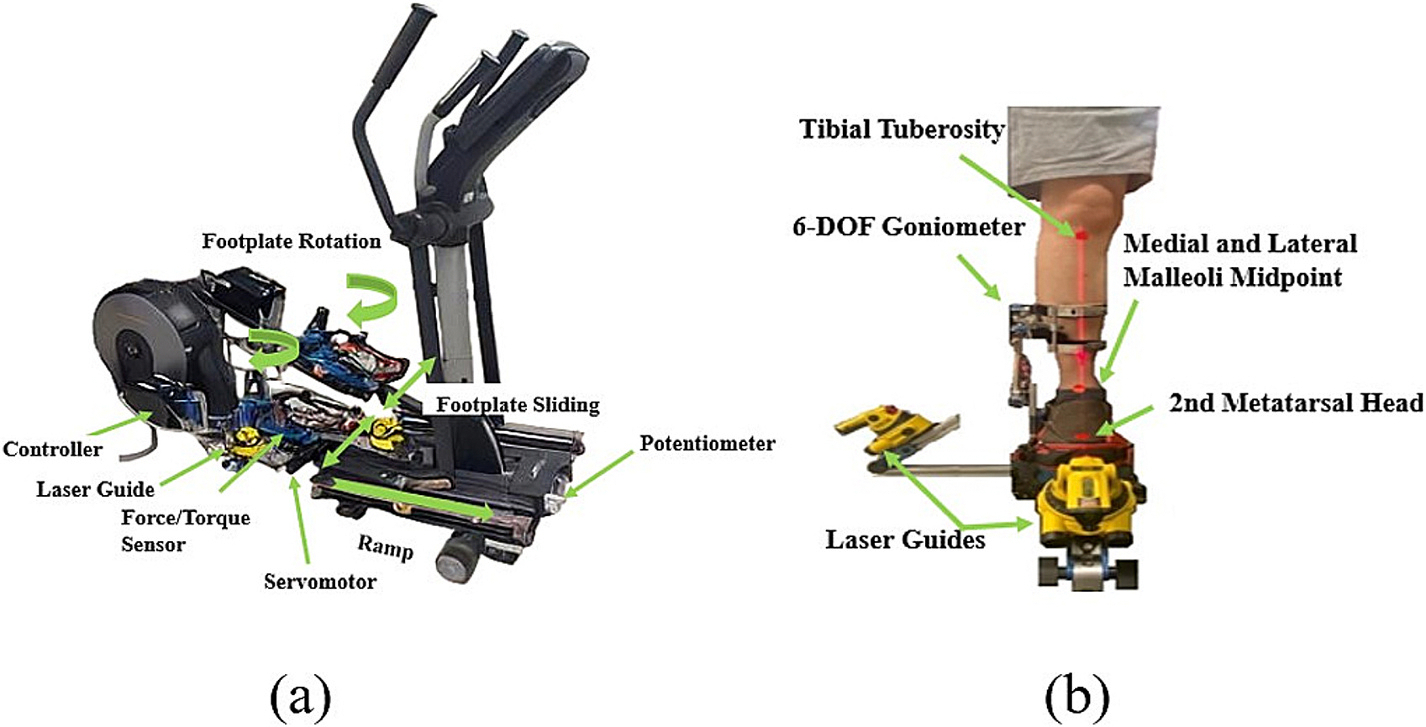
(a) Robotic off-axis stepping system. (b) Calibration of anatomical ankle zero angles. In the frontal plane, the force/torque sensor’s center, the second metatarsal head, the midpoint between the medial and lateral malleoli, and the tibia tuberosity were aligned using laser alignment guides. In the sagittal plane, the force/torque sensor’s center, the lateral malleolus, and the peripheral margin of the lateral tibial plateau were aligned using laser alignment guides.

A robotic off-axis stepping system with multidirectional footplate movement in the frontal and transverse planes, providing a controlled environment for targeted off-axis stepping training, was used in this study (Kang et al., 2014; Kang et al., 2019; Lee et al., 2020; Ren et al., 2013) (Fig. 1a). The device’s footplates were controlled to rotate in the transverse plane and slide in the frontal planes, respectively, facilitating optimal stepping positions. Three-dimensional (3-D) tibia kinematics were measured using a compact six-degree-of-freedom goniometer, affixed firmly to the lower leg’s bony anteromedial aspect and connected distally to the footplate (Fig. 1b). The 3-D footplate reaction forces and torques were measured using a six-axis force/torque sensor (JR3, Woodland, CA, USA) mounted beneath each footplate (Fig. 1a). Participants’ feet were strapped to the footplates to ensure stable foot positioning on the footplates during stepping.

A standing position with a tibial inclination angle of 0 degrees in all 3 dimensions, including anterior/posterior inclinations, medial/lateral inclinations, and internal/external rotation inclinations, was established on the stepping system using laser alignment tools to align the peripheral margin of the lateral tibial plateau, the lateral malleolus, and the center of the force/torque sensor in the sagittal plane and align the tibial tuberosity, the mid-point between the lateral and medial malleoli, the second metatarsal head, and the center of the force/torque sensor in the frontal plane (Fig. 1b). Participants’ anthropometric data, tibia kinematics, and forces and moments measured at the footplate were used in a custom inverse dynamics algorithm to determine the 3-D knee moments in real-time (Kang et al., 2014; Kang et al., 2019; Lee et al., 2020; Ren et al., 2013). The reliability and validity of the real-time knee moment estimation method was established in a previous study using comparing the results of this method with an offline method (Kang et al., 2013).

Participants were asked to wear comfortable exercise clothing and their self-selected athletic footwear. They were first familiarized with the stepping device, after which they engaged in a two-minute stepping session at their self-selected speed without tightly gripping the support handles. No resistance was used for the elliptical stepping, which was kept identical for all participants. The stepping conditions were varied through gradual adjustments of the footplates from narrow (NrSW, 23 cm) to neutral (NtSW, 28 cm) and to wide (WdSW, 35 cm) step-widths. Our study considered the horizontal distance between the center of the two footplates as the step-width. After participants completed a minimum of ten consistent stepping cycles at each target step-width, the footplates were smoothly transitioned to the next predetermined step-width position while the participants stepped continuously in the process. The robot-controlled footplate displacement between these widths during stepping was smooth and slow (about 0.5 mm/s), and the participants were unaware of the footplate mediolateral sliding. The footplate transition was performed simultaneously on the right and left footplates. However, the measurements and analysis were focused on the right side.

### 2.3. Data processing

Data analysis was performed on ten consecutive stepping cycles for each experimental condition. A complete cycle was defined as the motion of the right foot from its foremost position back to the same point. The elliptical stepping cycle was normalized to a percentage scale ranging from 0 % to 100 %. The analysis focused on the initial 50 % of the stepping cycle representing the loading phase. During this phase, the external knee moment (EKM) components, namely the knee flexion moment (KFM), the knee adduction moment (KAM), and the knee internal rotation moment (KIRM) were normalized to the product of body weight and height, expressed as a percentage (%(BW*HT)). The maximum value of each EKM component during the loading phase was determined. The knee adduction moment impulse (ImpKAM) was calculated by integrating the KAM over the loading stance phase. Additionally, the 3-D tibia inclination angles and 3-D footplate reaction forces corresponding to the pKAM time were identified. Footplate reaction forces were normalized to the body weight and expressed as a percentage (%(BW)). For each participant, the average values of peak knee flexion moment (pKFM), peak knee adduction moment (pKAM), peak knee internal rotation moment (pKIRM), ImpKAM, tibia inclination angles, and footplate reaction forces were calculated from the ten cycles.

### 2.4. Statistical analysis

We employed specific statistical tests to explore the impact of different step-widths on pKFM, pKAM, pKIRM, and ImpKAM. A repeated-measures analysis of variance (RM-ANOVA) was used to evaluate the main effect for step-width (NrSW, NtSW, and WdSW) as the within-subject factor and group (KOA and age-gender matched control) as the between-subject factor for each variable. Post-hoc tests using a Bonferroni correction were used to identify significant differences in cases of significant main or interaction effects. The difference in the stepping speed, reported as revolutions per minute (rpm), between the different stepping conditions was also assessed.

A forward stepwise multiple linear regression analysis was conducted to explore the variance in pKAM explained by the presence of KOA, tibia inclination angles, and footplate reaction forces/moments corresponding to the pKAM time during stepping. While controlling for age, sex, and stepping speed, the following covariates were included in a forward stepwise multiple regression model (*P*_*in*_ = 0.05, *P*_*out*_ = 0.1): group (KOA and healthy control), tibia anterior inclination, tibia medial inclination, tibia internal rotation inclination, footplate reaction force in the lateral, anterior, and vertical directions, footplate-reaction dorsi flexion torque, inversion torque, and internal rotation torque. We verified the homoscedasticity of residuals using both the Breusch-Pagan and Koenker tests, and multicollinearity among predictors was evaluated using the variance inflation factor (VIF), with all values falling below the threshold of concern (VIF < 10).

To determine the rate of pKAM change during stepping with a gradually increasing step-width from NrSW to WdSW, the pKAM of each stepping cycle during the footplate transition from NrSW to WdSW was computed. The slope and intercept of the linear relationship between the pKAM and step-width, representing the rate of pKAM change with step-width and the pKAM at the NtSW (also neutral step-width), respectively, were determined for each participant. This slope was compared to zero using the One-Sample Wilcoxon Signed Rank test. The intercept and slope were compared between the two groups. For all statistical tests, the significance level was set at 0.05. Before analysis, the normality of the data was assessed with a Shapiro-Wilk test. For comparisons involving non-normally distributed data, Friedman’s two-way analysis of variance was used for within-group comparisons. For the between-group comparison, an independent-sample Mann-Whitney test was used. These statistical evaluations were processed using SPSS Version 29 (IBM, Armonk, NY, USA). All values are reported as mean (standard deviation).

## 3. Results

### 3.1. Stepping speed and pKAM

There was no significant main effect of step-width on stepping speed, F(2, 52) = 0.118, *P* = 0.889. No between-group difference was found between the stepping speed across all three conditions (for the KOA group; NrSW:29.92 ± 8.24 rpm, NtSW:29.78 ± 8.17 rpm, WdSW:30.45 ± 7.74 rpm, and for the control group; NrSW:33.20 ± 4.79 rpm, NtSW:33.00 ± 5.34 rpm, WdSW:33.95 ± 5.84 rpm) (*P* = 0.204).

**Fig. 2.**
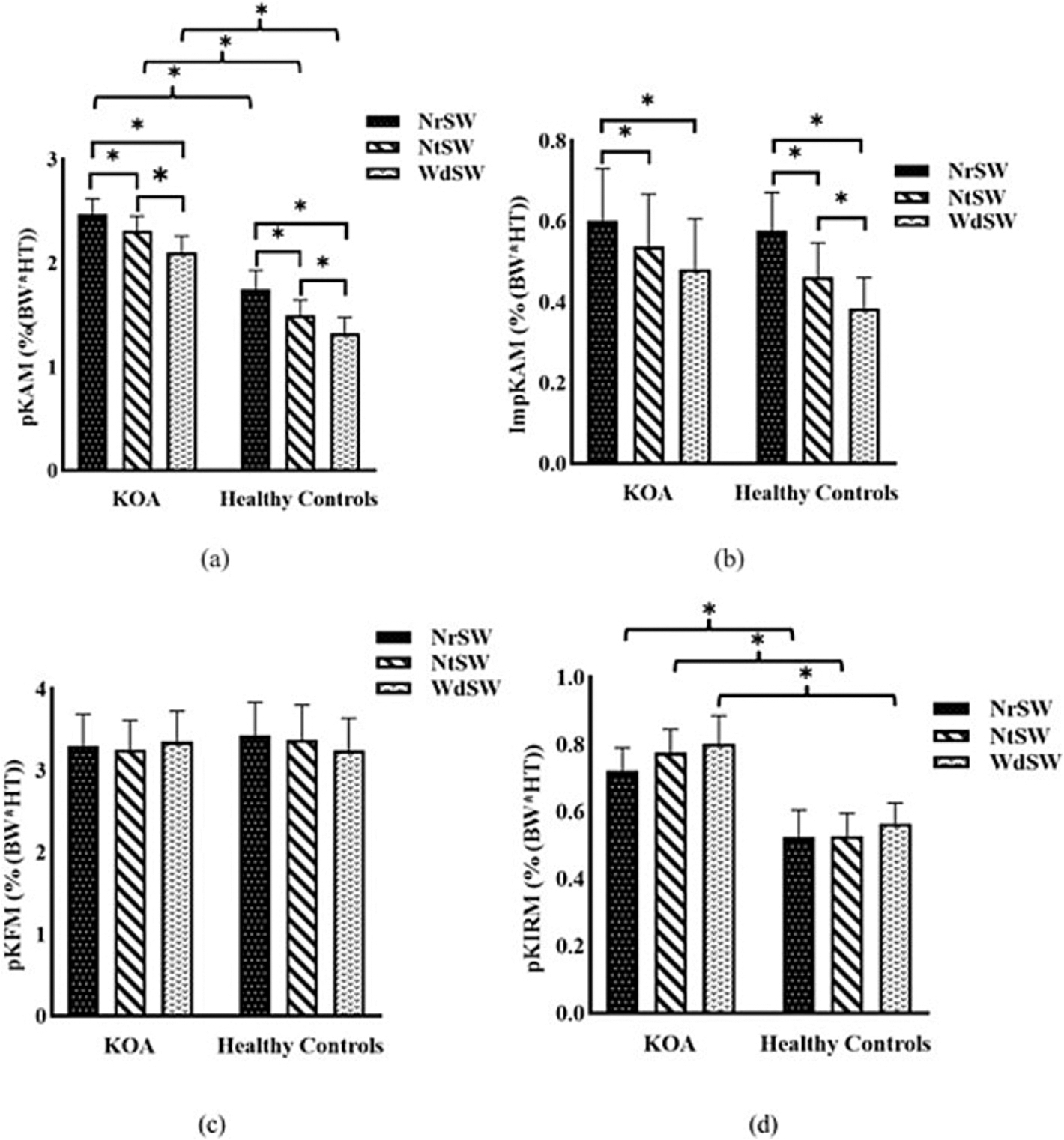
(a) peak knee adduction moment (pKAM) and (b) knee adduction moment impulse (ImpKAM) during stepping with different step-widths in KOA and healthy control groups. NrSW: Stepping with the narrowest distance between the footplates. (c) peak knee flexion moment (pKFM) and (d) peak knee internal rotation moment (pKIRM) during stepping with different step-widths in KOA and healthy control groups. NrSW: Stepping with the narrowest distance between the footplates. NtSW: Neutral stepping with no change in the footplate Neutral distance, WdSW: Stepping with the widest distance between the footplates. Error bars represent the standard error. * *p* < 0.05.

A significant main effect of step-width on the pKAM was found (F (1.33, 36.18) = 27.323, *P* < 0.001, partial η2 = 0.512). The KOA group showed higher pKAM values across all stepping conditions (NrSW: 2.47 ± 0.56 vs 1.74 ± 0.69 %BW*HT, *P* = 0.005; NtSW: 2.31 ± 0.53 vs 1.50 ± 0.55 %BW*HT, *P* < 0.001; WdSW: 2.10 ± 0.59 vs 1.32 ± 0.59 % BW*HT, *P* = 0.002). In both groups, WdSW produced lower pKAM compared to NtSW (KOA: *P* = 0.008; Control: *P* = 0.030) and NrSW (KOA: *P* = 0.003; Control: *P* < 0.001). Additionally, NtSW resulted in lower pKAM than NrSW (KOA: *P* = 0.037; Control: *P* < 0.001) (Fig. 2a).

### 3.2. ImpKAM

A significant main effect of step-width on the ImpKAM was revealed (F(1.430, 37.173) = 29.304, *P* < 0.001, partial η^2^ = 0.530). Both groups showed lower ImpKAM in NtSW (KOA: 0.54 ± 0.48, Control: 0.46 ± 0.31 %BW*HT*s) compared to NrSW (KOA: 0.60 ± 0.49, Control: 0.58 ± 0.35 %BW*HT*s; *P* = 0.022 and *P* < 0.001, respectively). WdSW (KOA: 0.48 ± 0.47, Control: 0.38 ± 0.28 %BW*HT*s) also showed lower ImpKAM compared to NrSW (*P* = 0.009 and *P* < 0.001, respectively). In healthy controls only, NtSW induced lower ImpKAM than WdSW (*P* = 0.016) (Fig. 2b).

### 3.3. pKFM

No significant main effect of step-width was found on pKFM (F (1.680, 43.668) = 0.377, *P* = 0.652, partial η2 = 0.014), with similar values across all conditions for both KOA (NrSW: 3.30 ± 1.45, NtSW: 3.25 ± 1.35, WdSW: 3.36 ± 1.39 %BW*HT) and control groups (NrSW: 3.43 ± 1.51, NtSW: 3.38 ± 1.58, WdSW: 3.25 ± 1.48 %BW*HT). No significant difference was found between groups (F (1, 26)=0.008, *P* = 0.928, partial η2 < 0.001) (Fig. 2c).

### 3.4. pKIRM

Friedman’s analysis showed no significant difference in pKIRM between step-widths for either the KOA (*P* = 0.319) or control group (*P* = 0.17). However, the KOA group demonstrated higher pKIRM values across all conditions (NrSW: 0.72 ± 0.26 vs 0.52 ± 0.30 %BW*HT, *P* = *0.035;* NtSW: 0.78 ± 0.26 vs 0.80 ± 0.31 %BW*HT, *P* = 0.009; WdSW: 0.80 ± 0.31 vs 0.57 ± 0.22 %BW*HT, *P* = 0.021) (Fig. 2d).

### 3.5. Slope and intercept analysis

**Fig. 3.**
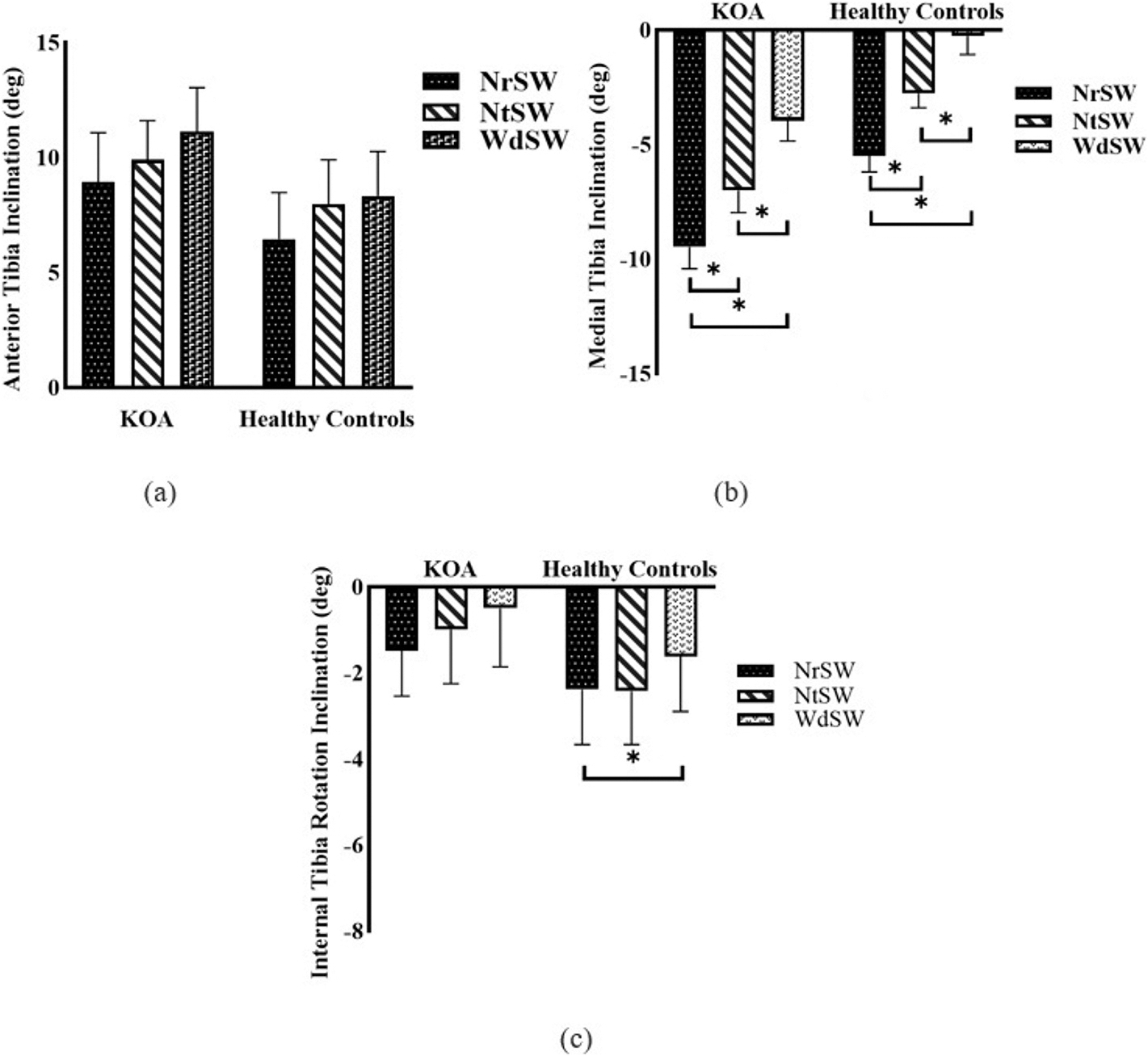
Rate of peak knee adduction moment (pKAM) change with continuous step-width change indicated as slope, and pKAM at neutral footplate position (intercept) in KOA (*n* = 14) and age and gender-matched healthy controls (n = 14) during footplate transition from NrSW to WdSW.

Comparison of the slope of the pKAM-step width relationship to zero showed that for both the KOA and healthy control groups slope was lower than zero (*P* = 0.019, *P* = 0.016, respectively). Furthermore, the slope and the intercept of the KOA group were higher than that of the healthy control group (*P* < 0.01 and *P* < 0.001, respectively) (Fig. 3). The slope and intercept for the KOA group were − 0.01 ± 00.01 (% (BW*HT)/mm) and 2.25 ± 0.60 (%(BW*HT)), respectively, and were − 0.001 ± 0.003 (%(BW*HT)/mm) and 0.22 ± 0.48 (%(BW*HT)), respectively for the healthy controls.

### 3.6. Tibia inclination angles

**Fig. 4.**
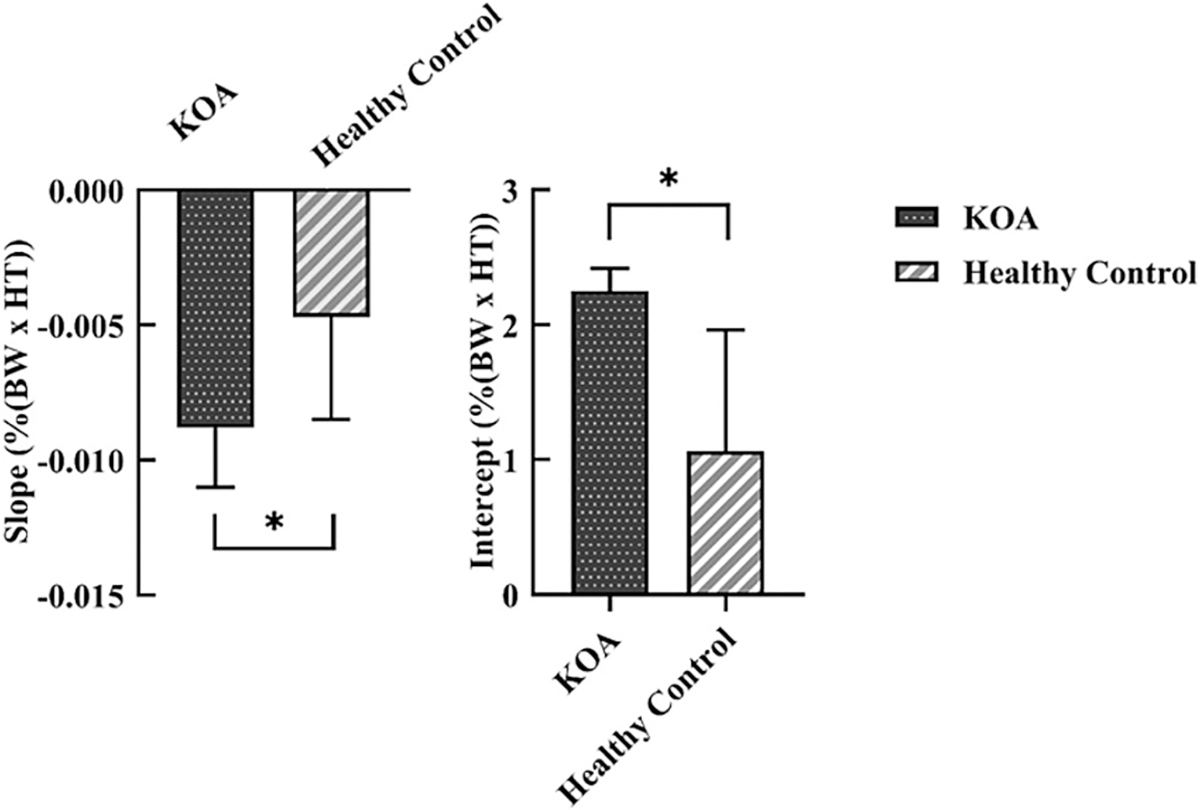
(a) Anterior tibia inclination, (b) medial tibia inclination, and (c) internal tibia rotation inclination angles corresponding to the peak knee adduction moment (pKAM) timing during stepping with different step-widths in KOA and healthy control groups. NrSW: Stepping with the narrowest distance between the footplates, NtSW: Neutral stepping with no change in the footplate Neutral distance, WdSW: Stepping with the widest distance between the footplates. Error bars represent the standard error. * p < 0.05.

Step-width significantly affected anterior tibia inclination (F(1.497, 38.926) = 4.412, *P* = 0.028, partial η^2^ = 0.145), with differences between NtSW and WdSW (*P* = 0.022) when groups were combined. The KOA group showed values of 8.94 ± 7.99°, 9.91 ± 6.35°, and 11.14 ± 7.14° for NrSW, NtSW, and WdSW, respectively, while the control group showed 6.45 ± 7.57°, 7.98 ± 7.23°, and 8.33 ± 7.26°. However, within-group pairwise comparisons showed no significant differences (all *P* > 0.05) (Fig. 4a).

Medial tibia inclination increased with step-width in both groups (F (1.589, 41.323) = 212.712, *P* < 0.001, partial η^2^ = 0.891). The KOA group values were − 9.47 ± 3.55°, −7.01 ± 3.61°, and − 3.96 ± 3.32° for NrSW, NtSW, and WdSW respectively, while the control group showed −5.50 ± 2.60°, −2.76 ± 2.39°, and − 0.25 ± 3.03°, showing significant differences between all conditions (all *P* < 0.001). The control group demonstrated higher medial tibia inclination across all conditions (NrSW: *P* = 0.002; NtSW: *P* = 0.001; WdSW: *P* = 0.005) (Fig. 4b).

For tibia internal rotation inclination (F(1.506, 39.144) = 4.779, *P* = 0.022, partial η^2^ = 0.155), the KOA group showed values of −1.49 ± 3.93°, −2.39 ± 4.77°, and − 0.99 ± 4.72° for NrSW, NtSW, and WdSW respectively, while the control group showed −2.43 ± 4.64°, −0.50 ± 5.10°, and − 1.62 ± 4.77°. Only the control group showed higher values in NrSW compared to WdSW (*P* = 0.021) (Fig. 4c).

### 3.7. Footplate reaction forces

A significant main effect of step-width on the footplate lateral reaction force (F(1.121, 29.145) = 53.422, *P* < 0.001, partial η^2^ = 0.673). Both groups showed decreased lateral force with increased step-width, with significant differences between NtSW and NrSW (both *P* < 0.001), WdSW and NrSW (both *P* < 0.001), and WdSW and NtSW (KOA: *P* < 0.001; Control: *P* = 0.004). The KOA group values progressed from 1.32 ± 2.04 %BW in NrSW to −0.84 ± 2.12 %BW in NtSW and − 2.61 ± 3.28 %BW in WdSW, while the control group showed a similar pattern from 0.43 ± 1.50 %BW to −1.35 ± 7.29 %BW and − 3.66 ± 9.51 %BW, respectively.

For anterior force, the KOA group showed values of −5.11 ± 5.31 % BW, −3.12 ± 4.59 %BW, and − 3.05 ± 4.43 %BW for NrSW, NtSW, and WdSW, respectively, while the control group showed −6.57 ± 1.72 % BW, −4.79 ± 6.66 %BW, and − 4.95 ± 9.91 %BW.

Internal rotation force values for the KOA group were 0.60 ± 0.49 % BW, 0.54 ± 0.48 %BW, and 0.48 ± 0.47 %BW for NrSW, NtSW, and WdSW, respectively, while the control group showed 0.58 ± 0.35 %BW, 0.46 ± 0.31 %BW, and 0.38 ± 0.28 %BW.

### 3.8. Regression analysis

A forward stepwise regression analysis revealed that the following variables were significant predictors of pKAM in the final model: medial tibia inclination (*P* < 0.001), presence of KOA (*P* < 0.001), footplate reaction lateral force (*P* = 0.023), footplate reaction inversion torque (*P* < 0.001), and stepping speed (*P* = 0.022). This regression model explained 74.4 % of the variance in pKAM (R^2^ = 0.744, Adjusted R^2^ = 0.728). The tolerance and VIF values for all predictors in the final model were within acceptable ranges (Tolerance>0.1, VIF < 10), suggesting no multicollinearity issues (Table 2).

## 4. Discussion

This study explored changes in peak knee moments and knee adduction moment impulse over a range of step-widths in individuals with medial KOA and age and gender-matched healthy controls using a robotic off-axis stepping system. We specifically examined how the pKAM changes as the footplates move slowly from an NrSW of 23 cm to a WdSW of 35 cm. The findings supported our first and second hypotheses: we observed decreased pKAM and ImpKAM with increased step-width in both groups, while pKFM and pKIRM remained unchanged. Additionally, pKAM was consistently higher in KOA patients than healthy controls across all step-widths. The observed linear relationship between pKAM and step-width during smooth footplate transitions supports the feasibility of determining subject-specific step-width that minimizes pKAM to an optimal point.

Biomechanically, wider step-width brings the knee joint center closer to the foot’s center of pressure, reducing the external lever arm and thereby decreasing pKAM (Anderson et al., 2018). Our study showed that stepping with a wider step-width increased medial tibia inclination, which reduced pKAM by more valgus knee alignment (Table 2). A lower medial tibia inclination (i.e., less knee valgus) during stepping with NrSW may offer another potential application of our robotic elliptical system. Other populations, such as individuals with patellofemoral pain or lateral KOA, may be present with altered frontal plane biomechanics and may benefit from training with the robotic off-axis stepping system. Based on the results of the regression model, medial tibia inclination was the strongest predictor of pKAM, with each standard deviation (SD) increase associated with a 0.83 SD decrease in pKAM (Table 2). Additional predictors included footplate lateral reaction force (0.22 SD decrease in pKAM), footplate-reaction inversion torque (0.24 SD decrease in pKAM), and stepping speed (0.17 SD decrease in pKAM) (Table 2). These findings offer potential parameters that can be manipulated to reduce pKAM during elliptical stepping.

Our observed reductions in pKAM with wider step-widths, which were 6.13 ± 9.43 % (NtSW) and 14.35 ± 19.10 % (WdSW) in the KOA group, and 12.32 ± 11.07 % (NtSW) and 23.42 ± 20.96 % (WdSW) in controls, are consistent with previous findings from overground walking studies (Anderson et al., 2018; Byrnes et al., 2022; Favre et al., 2016). Stief et al. explored the effect of subject-specific step-width on KAM by comparing halved, doubled, and tripled step-width with normal step-width during gait (Stief et al., 2021). These authors reported a 9 % pKAM reduction with double step-width and 11 % with triple step-width compared to habitual gait. Their step-width conditions ranged from 6.9 cm (halved) to 30.1 cm (tripled), while typical overground walking step-widths range from 7 to 12 cm (Helbostad and Moe-Nilssen, 2003; Hollman et al., 2011; Wert et al., 2010). While previous studies, such as those by Stief et al. (2021), used discrete conditions (e.g., halved, doubled, and tripled step-widths) to assess pKAM, our approach enables continuous variation in step-width, providing more precise control over gait modification (Stief et al., 2021). Additionally, our use of a robotic off-axis stepping system allows for real-time measurement of knee moments, which is not only clinically practical but also provides insights into the most effective, individualized step-width for pKAM reduction. This methodological difference allows for a finer resolution in evaluating the subject-specific response to gait modification, which has not been addressed in prior research.

Importantly, prior studies have not clearly established a threshold for clinically meaningful pKAM reduction. However, Miyazaki et al. (2002) showed that a 1 % decrease in pKAM was associated with more than sixfold reduction in the risk of KOA progression (Miyazaki et al., 2002), and Erhart et al. (2009) found that a 6.6 % reduction in pKAM was associated with meaningful improvements in knee pain (Erhart et al., 2009). The magnitude of pKAM reductions observed in our study falls within or exceeds these thresholds, suggesting potential clinical relevance.

In contrast to overground walking studies, which have been the primary focus of prior investigations, there remains limited research on how step-width modifications affect knee moments during pedal-type exercise. One study using a non-motorized stepper (Pinnacle Trainer) reported increased internal knee adduction moment (corresponding to lower external knee adduction moment) with wider inter-pedal distance (You et al., 2019). On the contrary, another study that assessed the effects of 4 different inter-pedal distances of stationary cycling on the internal knee abduction moment found an increase in the internal knee abduction moment (e.g., external knee adduction moment) (Thorsen et al., 2020). This increase in KAM was attributed to the lack of a significant shift in the center of mass (COM) during cycling, which has been established as a contributing factor to the reduction of KAM with wider step-width during walking. Additionally, Stief et al. reported an increase in the external rotation of the foot with wider step-width, which was observed as an increase in the tibia internal rotation inclination for the healthy controls in our study. Collectively, our findings and the studies mentioned above confirm the dependence of pKAM on the step-width.

Examination of the pKFM revealed that pKFM, a major contributing factor to medial knee loading, did not significantly change with WdSW stepping. During gait, changes in ankle dorsi flexion can affect KFM (Landin et al., 2015) by modulating the GRF lever arm relative to the knee joint center. In our study, anterior tibia inclination, an equivalent measure of ankle dorsi flexion, did not change significantly during stepping with different step-widths. Therefore, the footplate reaction force lever arm in the sagittal plane did not differ statistically between these three stepping conditions. Furthermore, the vertical footplate reaction force, another factor that affects pKFM (Elhafez et al., 2019; Kluger et al., 2014), did not differ significantly between stepping conditions.

Like pKFM, the pKIRM did not change with a wider step-width. Our results showed that increasing step-width did not change the tibia internal rotation inclination angle, which is a contributing factor to the pKIRM. The unchanged pKFM and pKIRM during WdSW stepping suggests that total knee moment remains constant, making this approach promising for KOA rehabilitation, particularly when both medial compartment and patellofemoral OA are present.

The slope of the linear relationship between pKAM and step-width was used to quantify the rate of change in pKAM as a function of step-width. This slope provides valuable information on the magnitude of step-width modification required to achieve a desired reduction in pKAM in real-time within a single session. The advanced algorithm for real-time estimation of pKAM during footplate sliding offers a rapid and precise determination of the step-width, eliminating the need to evaluate multiple discrete step-widths. Moreover, this innovative and user-friendly system allows clinicians to customize the step-width based on the severity of varus knee alignment and KOA progression. This evaluation can be performed in a single trial and with simple post-processing to find the slope and desired step-width. The average slope for the normalized pKAM was −0.01 (%(BW*HT)/mm) for the KOA group and − 0.001 (%(BW*HT)/mm) for healthy controls (Fig. 3). The negative slope indicates that the wider the step-width, the lower the pKAM. The significantly higher slope in the KOA group, compared to the healthy controls, demonstrates a greater pKAM reduction per mm of increased step-width. The observed difference in the response of each group to the changes in the step-width during stepping emphasizes that researchers and clinicians should appreciate that an observation made in healthy individuals should not be generalized to individuals with KOA. The relatively small slope values suggest that the current inter-footplate distance range of the off-axis elliptical may not be sufficient to achieve an optimal pKAM reduction of 10 % for everyone. However, due to the mechanical limitations of the system, further increasing the step-width may only be feasible with some hardware modifications. Therefore, we propose investigating the combined effects of modifying FPA and step-width on pKAM. This strategy may result in greater pKAM reductions with smaller footplate adjustments for both FPA and step-width. Accordingly, FPA modifications, combined with the currently available inter-footplate distance, may be adequate for clinically meaningful pKAM reduction.

Although the current study was conducted on a robotic elliptical trainer, a similar pattern for the reduction in pKAM by 9–28 % by doubling and tripling step width during overground walking was observed (Xu et al., 2020). In clinical settings, step-width modifications during walking could be guided using treadmill-based feedback or wearable sensors to reduce pKAM. Xu et al. (2020) used a real-time feedback system on a treadmill, allowing 15 healthy individuals and one KOA patient to select gait patterns within a LOWESS-predicted “target gait zone” for KAM reduction. Seven chose increased step width, alone or with foot progression angle changes. However, they did not isolate step width’s specific effect on pKAM. Integrating their mapping approach with our subject-specific analysis may help with developing a tool for estimating the individualized relationship between pKAM and step width during overground walking and potentially translate elliptical-based findings to real-world gait training. Future studies are warranted to validate the transferability of these findings to natural gait and to determine whether longitudinal training intervention that employs individualized step-width retraining leads to long-term biomechanical and symptomatic benefits.

Several limitations of this study must be considered. Firstly, since the primary aim of this study was to preliminarily examine the feasibility of using a continuously adjustable robotic system for individualized determination of step-width, we used a small sample size. Therefore, we recommend caution in generalizing the findings to individuals with KOA. Further large-scale studies are needed to validate and extend these findings to broader populations. Secondly, our study did not examine lower limb muscle activity changes following step-width modifications. Given that step-width adjustments may influence medial knee muscle co-contraction, which is typically more pronounced in individuals with medial KOA than in asymptomatic individuals (Al-Khlaifat et al., 2016; Hodges et al., 2016), assessing muscular activity changes could provide a more comprehensive understanding of the biomechanical complexities of step-width modifications. Thirdly, in this study, we did not investigate the combined effects of various footplate modification strategies, including alterations in step-width along with FPA. Integrating these strategies might offer a more effective reduction in pKAM and ImpKAM, potentially requiring less dramatic changes in step-width. Lastly, the efficacy of subject-specific step-width modification in reducing pKAM must be further investigated through a long-term training program.

## 5. Conclusion

The robotic off-axis stepping trainer facilitates the determination of the relationship between pKAM and step-width, which can be utilized to guide subject-specific training interventions. The characteristics of this relationship allow for the identification of the minimum step-width that reduces pKAM by a clinically meaningful amount without an exaggerated step-width modification. Specifically, by employing this methodology, clinicians can reliably determine the optimal training strategy for their patients in real-time, eliminating the need for evaluating multiple discrete voluntary step-width adjustments, which may alter gait and knee joint loading patterns.

## Figures and Tables

**Table 1:** Participants characteristics.

Characteristics	KOA (n = 14)	Healthy control (n = 14)	P value

Age (years)	63.97 (10.11)	61.37 (15.00)	0.310
Height (m)	1.67 (0.07)	1.70 (0.10)	0.392
Mass (kg)	87.49 (16.41)	72.09 (13.52)	0.280
BMI (kg.m−2)	31.95 (7.10)	25.02 (3.97)	0.112
Female (number (%))	12 (85.71)	12 (85.71)	–
Male (number (%))	2 (14.29)	2 (14.29)	–
KOOS Pain	68.06 (19.80)	–	–
KOOS ADL	74.73 (19.01)	–	–
Kellgren Lawrence	2.18 (0.87)	–	–

**Table 2:** The covariates of a forward-stepwise regression model explaining the pKAM changes with a change in the step width (*n* = 28).

Covariate	β (standardized)	P value	Adjusted r2	VIF

Tibia Medial Inclination (°)	−0.834	<0.001	0.63	3.11
Presence of OA	−0.267		0.67	1.72
Footplate Reaction Lateral Force (%(BW))	−0.221	0.023	0.69	2.78
Footplate Reaction Inversion Torque (%(BW*HT))	0.244	<0.001	0.71	1.36
Stepping Speed (rpm)	0.165	0.022	0.73	1.53

The regression model was controlled for age, sex, and stepping speed.

a *p*-value <0.05 was considered statistically significant.

VIF: variation inflation factor.

rpm: revolution per minute.
